# Quantitative Evaluation and Obstacle Factor Diagnosis of Agricultural Drought Disaster Risk Using Connection Number and Information Entropy

**DOI:** 10.3390/e24070872

**Published:** 2022-06-25

**Authors:** Yi Cui, Juliang Jin, Xia Bai, Shaowei Ning, Libing Zhang, Chengguo Wu, Yuliang Zhang

**Affiliations:** 1 School of Civil Engineering, Hefei University of Technology, Hefei 230009, China; ycui@hfut.edu.cn (Y.C.); 2021800032@hfut.edu.cn (X.B.); ning@hfut.edu.cn (S.N.); zhanglibing77@gmail.com (L.Z.); wucguo82@gmail.com (C.W.); zhangyuliang@hfut.edu.cn (Y.Z.); 2 Institute of Water Resources and Environmental Systems Engineering, Hefei University of Technology, Hefei 230009, China

**Keywords:** drought disaster risk evaluation, obstacle factor diagnosis, information entropy, evaluation sample information, connection number, structural water resources science, Suzhou City

## Abstract

To promote the application of entropy concepts in uncertainty analysis of water resources complex system, a quantitative evaluation and obstacle factor diagnosis model of agricultural drought disaster risk was proposed using connection number and information entropy. The results applied to Suzhou City showed that the agricultural drought disaster risks in Suzhou during 2007–2017 were all in middle-risk status, while it presented a decreasing trend from 2010. The information entropy values of the difference degree item *bI* were markedly lower than those of the difference degree *b*, indicating that *bI* provided more information in the evaluation process. Furthermore, the status of drought damage sensitivity and drought hazard were improved significantly. Nevertheless, high exposure to drought and weak drought resistance capacity seriously impeded the reduction of risk. Thus, the key to decreasing risk was to maintain the level of damage sensitivity, while the difficulties were to reduce exposure and enhance resistance. In addition, the percentage of the agricultural population, population density, and percentage of effective irrigation area were the main obstacle factors of risk and also the key points of risk control in Suzhou. In short, the results suggest that the evaluation and diagnosis method is effective and conducive to regional drought disaster risk management.

## 1. Introduction

Food security is a hot topic that arouses constant attention from the international community [1,2]. With the increasing global climate change and the impact of human activities, the frequency, intensity, and scope of influence of drought increase significantly; thus, the drought disaster risk continues to increase. Drought disaster has seriously threatened food security in many countries and regions [3,4]. China is located in the southeast of Eurasia, characterized by a remarkable monsoon climate, uneven spatial and temporal distribution of precipitation, and frequent agricultural drought disasters [5,6,7]. According to the statistics, the annual average drought-affected area in China from 2006 to 2020 is 1.39 × 10^7^ hectares, causing grain losses of 2.14 × 10^10^ kg and direct economic losses of RMB 8.19 × 10^9^ billion [8]. Agricultural drought disaster has become one of the severe challenges facing food security and rural revitalization in China [9,10]. Since the 21st century, with the transformation from passive drought relief and emergency management to active drought relief and risk management, drought disaster risk management has gradually emerged [11,12,13]. Quantitative evaluation and obstacle factor diagnosis of drought disaster risks are the core contents and key points of risk management [14]. Therefore, the accurate evaluation and diagnosis of agricultural drought disaster risk in China’s main grain production areas are of great significance for improving the human ability to cope with droughts and providing guidance for regional drought disaster risk management [15,16].

Drought disaster risk evaluation is a hot spot in the field of natural disaster research. The existing methods mainly include three kinds [17]: (1) A probability statistics-based method. First, the great work of Kolmogorov [18] laid the foundation of probability and stochastics, which realized the axiomatization of probability theory. Koutsoyiannis et al. [19] pointed out that modern statistical and quantum physics promoted a new understanding and modeling of physical processes, such as drought disaster risk formation. Hao et al. [20] built a drought disaster loss frequency analysis model based on historical data and information diffusion theory and analyzed the agricultural drought disaster risk. Koutsoyiannis [21] proposed a general methodology for more theoretically justified stochastic processes, which provided an important thought for drought disaster risk evaluation from a probability statistics aspect. Xie et al. [22] used information diffusion and information matrix to analyze the probability and distribution of agricultural drought disaster risk. Yu et al. [23] established a natural disaster risk evaluation model composed of an evaluation index, relative loss calculation, and information diffusion. (2) A system-theory-based method. Kim et al. [24] built drought hazard index and vulnerability index, respectively, and multiplied these two to obtain the drought disaster risk index. Luo et al. [25] established a drought disaster risk evaluation system from four aspects and proposed a panel-data-based grey cloud clustering evaluation model. Hoque et al. [26] presented a comprehensive evaluation method for agricultural drought disaster risk by fuzzy superposition of all risk elements. (3) A formation cause-based method. Zhang et al. [27] used the CERES model to fit the relationship between the physical vulnerability curve of crops and the drought hazard index and established a dynamic evaluation model for drought disaster risk of maize. Wang et al. [28] adopted the APSIM model to establish the relationship between the frequency of drought intensity and the percentage of crop yield loss and evaluated the drought disaster risk of spring maize. Yin et al. [29] used the GEPIC-Vulnerability–Risk model to assess the occurrence probability and likelihood of yield losses from drought and conducted a quantitative evaluation of drought disaster risk for global-scale maize. However, considering the connection and evolution among drought disaster risk elements, as well as the feasibility of evaluation principle, data, and model, the current most effective and most widely used method for quantitative evaluation of drought disaster risk is to select representative evaluation indexes from drought disaster risk system, and adopt a system comprehensive evaluation thought [17,30].

Under the combined influence of natural conditions and social economy, the drought disaster risk system is a typical complex system [31]. At present, the multi-index system comprehensive evaluation by establishing an evaluation system and model is still an effective drought disaster risk evaluation method [17]. However, most models fail to take the uncertainty between the evaluation sample and evaluation criteria into full consideration, resulting in the deviation of evaluation results. Set pair analysis is a new quantitative analysis method of uncertainty [32]. The identical–different–inverse relation structure of the connection number for the set pair composed of two sets, i.e., evaluation sample and evaluation criteria, can be used to fully reflect the certainty and uncertainty between these two sets, which has been widely applied to natural disasters risk evaluation [33]. Wang et al. [34] proposed a set pair analysis method for the evaluation of flood damage risk. Zou et al. [35] adopted a set pair analysis-variable fuzzy set model to evaluate flood risk. Chen et al. [36] used set pair analysis and connection entropy to conduct a quantitative evaluation and diagnosis of agricultural drought disaster resistance capacity. Ge et al. [37] conducted an environmental impact risk evaluation of dam break based on set pair analysis and cloud model. Lyu et al. [38] adopted interval fuzzy numbers and set pair analysis to establish an evaluation model for urban water quality risk. Qu et al. [39] coupled unascertained measurement evaluation and set pair analysis to assess the risk of limestone karst water pouring out from the floor. Nevertheless, when set pair analysis is adopted for comprehensive system evaluation, most studies only reflect the evaluation results by the components of the connection number; they cannot determine the complete connection number value, which may cause information loss and restrict the development of set pair analysis [40]. What is more, the calculation of the difference degree coefficient is the key to determining the connection number value. In addition, only a few studies have been conducted on the quantitative evaluation of agricultural drought disaster risk in grain production areas, let alone the diagnosis of risk obstacle factors based on set pair analysis.

As an important part of the connection number, the difference degree coefficient is used for describing the uncertainty of set pair at a micro level, which has a significant impact on research results [32]. Some scholars have conducted studies on the calculation of the difference degree coefficient. Li et al. [41] derived the difference degree coefficient from the target value of the connection number. Tang [42] proposed an expert estimation approach of the difference degree coefficient. Pan et al. [43] built a trapezoidal fuzzy number of the difference degree coefficient and determined its value range within a confidence interval. Li et al. [40] adopted a full and partial connection number to distribute the difference degree coefficient. However, most of the calculation methods of the difference degree coefficient are rough, and they can only obtain the approximate value or interval range, which may cause a deviation between the research result and the actual situation. Moreover, the difference degree coefficient should be calculated fully combined with the information provided by evaluation samples. Therefore, a reasonable method for determining the difference degree coefficient is urgently needed.

What is more, some key studies have been completed, which provide effective thoughts for drought disaster risk evaluation and diagnosis. Wang et al. [44] assessed the agricultural drought disaster risk based on the set pair analysis theory. Chen et al. [33] conducted the risk assessment of agricultural drought disasters using the improved connection number and entropy information diffusion. Jin et al. [30] adopted semipartial subtraction set pair potential of ternary connection number to evaluate the drought disaster risk and identify its influencing factors. However, the connection number value method combined with the dynamic difference degree coefficient has not been used in the field of drought disaster risk.

According to the analysis of the characteristics and problems of agricultural drought disaster risk in China’s main grain production areas, an agricultural drought disaster risk system is composed of four subsystems, i.e., drought hazard, drought damage sensitivity, exposure to drought, and drought resistance, as well as the evaluation grade criteria, were established, from the perspective of natural disaster risk system structure. Based on set pair analysis theory, a calculation method of dynamic difference degree coefficient varying with evaluation sample was used to determine the connection number value. In addition, the information provided by connection number components and connection number value in the evaluation process were compared according to the information entropy method. Then a quantitative evaluation and diagnosis model of agricultural drought disaster risk was established to assess the risk level and diagnose its key obstacle factors. Furthermore, an empirical study was conducted in Suzhou City of Anhui Province, China, to provide an important scientific basis for regional drought disaster risk regulation and control.

## 2. Materials and Methods

### 2.1. Agricultural Drought Disaster Risk Evaluation and Diagnosis Model Based on Connection Number and Information Entropy

**Figure 1:**
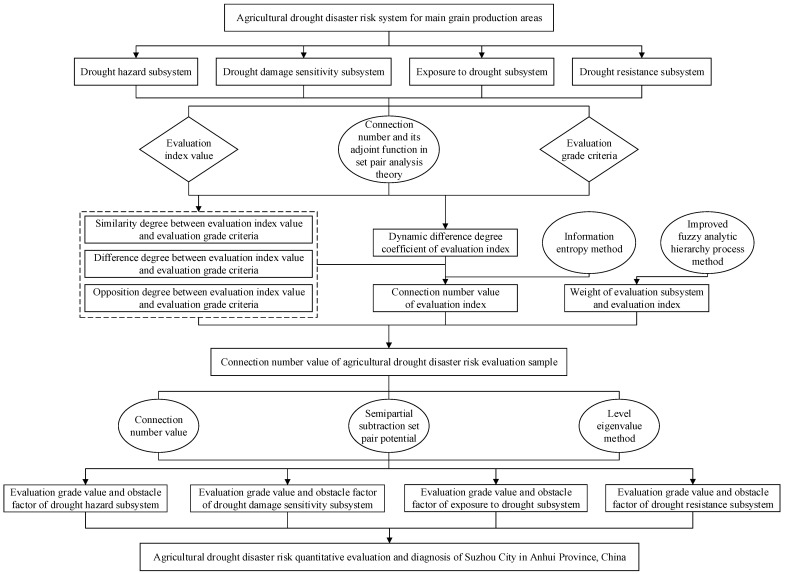
The establishment process of agricultural drought disaster risk evaluation and obstacle factor diagnosis model based on connection number and information entropy.

The establishment of the agricultural drought disaster risk evaluation and obstacle factor diagnosis model included the following steps, as shown in Figure 1. Firstly, an agricultural drought disaster risk system composed of four subsystems was built and based on connection number and its adjoint function, the similarity degree, difference degree, and opposition degree between evaluation index value and evaluation grade criteria were calculated. Then, combined with the proposed dynamic difference degree coefficient, the connection number value of each evaluation index was obtained. In addition, information entropy theory was used to compare the information provided by connection number value and connection number components in evaluation process. Finally, according to the weights of subsystem and evaluation index obtained by improved fuzzy analytic hierarchy process method, a quantitative evaluation and diagnosis model of agricultural drought disaster risk was established and applied to Suzhou City. Meanwhile, the results of semipartial subtraction set pair potential and level eigenvalue method were compared.

(1) According to the structure analysis and function analysis of agricultural drought disaster risk system composed of drought hazard, drought damage sensitivity, exposure to drought, and drought resistance [17], as well as the practical investigation, expert consultation, and reference statistics [30,44] in study area, an evaluation system of agricultural drought disaster risk {*x_kj_*|*k* = 1, 2, 3; *j* = 1, 2, …, *n_k_*} was established, and the corresponding sample set of index was described as {*x_ikj_*|*i* = 1, 2, …, *m*; *k* = 1, 2, 3; *j* = 1, 2, …, *n_k_*}. Among them, *x_ikj_* is the value of index *j* in subsystem *k* of evaluation sample *i*, *m* is the number of samples, *k* = 1, 2, 3, 4, which represents four subsystems, i.e., drought hazard, drought damage sensitivity, exposure to drought, and drought resistance, *n_k_* is the number of indexes in subsystem *k*. Without loss of generality, in this study, the drought disaster risk was divided into three grades, and the evaluation grade criteria were described as {*s_gkj_*|*g* = 1, 2, 3; *k* = 1,2, 3, 4; *j* = 1, 2,…, *n_k_*}, where *g* = 1, 2, 3, representing low, middle, and high risk, respectively.

(2) The improved fuzzy analytic hierarchy process based on accelerating genetic algorithm (AGA-FAHP) [45] was adopted to calculate the weights of each subsystem and each evaluation index {*w_kj_*|*k* = 1, 2, 3, 4; *j* = 1, 2, …, *n_k_*} as follows:

For a given agricultural drought disaster risk subsystem *k*, experts were invited to compare the importance of any two indexes in this subsystem for drought disaster risk so as to obtain the fuzzy complementary judgment matrix *A_k_* = (*a_kjl_*)*_nk_*_×_*_nk_*. AGA-FAHP was used to check and correct the consistency of *A_k_* and calculate the weight of each index, i.e., *w_kj_*. *A_k_* needed to be corrected if its consistency was unsatisfactory. Assume that the corrected matrix of *A_k_* was *B_k_* = (*b_kjl_*)*_n__k_*_×*n*_*_k_*. For simplicity, the sorting weight of elements in *B_k_* was still described as {*w_kj_*|*k* = 1, 2, 3, 4; *j* = 1, 2, …, *n_k_*}. Then, when the following formula [45]

(1) $$\min CIC\left(n_{k}\right)=\sum_{j=1}^{n_{k}}\sum_{l=1}^{n_{k}}\left|b_{jl}^{k}-a_{jl}^{k}\right|/n_{k}^{2}+\sum_{j=1}^{n_{k}}\sum_{l=1}^{n_{k}}\left|0.5\left(n_{k}-1\right)\left(w_{kj}-w_{kl}\right)+0.5-b_{jl}^{k}\right|/n_{k}^{2},\;s.t.\;\;\;\left\{ \begin{array}{l} b_{jj}^{k}=0.5\\ 1-b_{lj}^{k}=b_{jl}^{k}\in \left[a_{jl}^{k}-d,a_{jl}^{k}+d\right]\cap \left[0,1\right],\;\;\;\;\;k=1,2,3,4;j=1,2,\ldots ,n_{k};l=j+1,j+2,\ldots ,n_{k}\\ \sum_{j=1}^{n_{k}}w_{kj}=1.0,w_{kj}\in \left[0,1\right] \end{array}\right.$$

reached the minimum value, *B_k_* was called the optimal fuzzy consistency judgment matrix of *A_k_*, where *CIC*(*n_k_*) is the consistency index coefficient, *d* is a non-negative parameter within [0, 0.5]. Accelerating genetic algorithm (AGA) was a universal global optimization method, it was effective to solve the complex optimization problem in Formula (1). When *CIC*(*n_k_*) was smaller than a critical value, it indicated that *A_k_* had satisfactory consistency. Accordingly, the sorting weight of each evaluation index calculated was acceptable; otherwise, *d* needed to be adjusted until *A_k_* had satisfactory consistency. By a large number of numerical simulations and referring to related research [45], this study considered that when *CIC* was less than 0.20, the consistency of judgment matrix was regarded as satisfactory, and the obtained weight of each index was reasonable and acceptable.

(3) Set pair analysis (SPA) was a quantitative analysis theory of uncertainty proposed by Zhao Keqin in 1989 [32]. The foundation of SPA was the connection number *u* which was composed of two sets with a certain relation. Furthermore, the components of the connection number *u*, i.e., similarity degree *a*, difference degree *b*, and opposition degree *c,* were used to quantitatively describe the certainty and uncertainty of set pair from three aspects, i.e., identity, difference, and inverse, and *a* + *b* + *c* = 1. Specifically, SPA divided the certainty of set pair into identical status and inverse status, which were quantitatively described by *a* and *c*, respectively. Meanwhile, SPA divided the uncertainty of set pair between identical status and inverse status into the uncertainty at a macro level, measured by *b*, and that at a micro level, measured by difference degree coefficient *I*. Among them, the most commonly used ternary connection number *u* is expressed as follows [32]:

(2) u=a+bI+cJ

where *I* is the difference degree coefficient, whose value varies with the relation type of set pair. The set pair in this study belonged to positive–negative relation; the value of *I* ranged within [−1, 1]. *J* is the opposition degree coefficient and took −1 in this study [32].

Based on SPA and the practical problem of agricultural drought disaster risk evaluation, the evaluation index value and evaluation grade criteria constituted a set pair. The connection number of index was calculated according to the proximity between index value and grade criteria. Specifically, for two sets of evaluation index sample value of agricultural drought disaster risk *x_ikj_* and evaluation grade criteria *s_gkj_* (*g* = 1, 2, 3), the components of ternary connection number for index, i.e., *u_gikj_* are as follows [46]:

(3) $$u_{1ikj}=\{\begin{array}{l} 1,\;\;s_{0kj}\leq x_{ikj}\leq s_{1kj}\;\mathrm{for}\;\mathrm{positive}\;\mathrm{index},\;\;s_{0kj}\geq x_{ikj}\geq s_{1kj}\;\mathrm{for}\;\mathrm{negative}\;\mathrm{index}\\ 1-2\left(x_{ikj}-s_{1kj}\right)/\left(s_{2kj}-s_{1kj}\right),\;\;s_{1kj}<x_{ikj}\leq s_{2kj}\;\mathrm{for}\;\mathrm{positive}\;\mathrm{index},\;\;s_{1kj}>x_{ikj}\geq s_{2kj}\;\;\mathrm{for}\;\mathrm{negative}\;\mathrm{index}\\ -1,\;\;s_{2kj}<x_{ikj}\leq s_{3kj}\;\mathrm{for}\;\mathrm{positive}\;\mathrm{index},\;\;s_{2kj}>x_{ikj}\geq s_{3kj}\;\;\mathrm{for}\;\mathrm{negative}\;\mathrm{index} \end{array}$$

(4) $$u_{2ikj}=\{\begin{array}{l} 1-2\left(s_{1kj}-x_{ikj}\right)/\left(s_{1kj}-s_{0kj}\right),\;\;s_{0kj}\leq x_{ikj}\leq s_{1kj}\;\mathrm{for}\;\mathrm{positive}\;\mathrm{index},\;\;s_{0kj}\geq x_{ikj}\geq s_{1kj}\;\mathrm{for}\;\mathrm{negative}\;\mathrm{index}\\ 1,\;\;s_{1kj}<x_{ikj}\leq s_{2kj}\;\mathrm{for}\;\mathrm{positive}\;\mathrm{index},\;\;s_{1kj}>x_{ikj}\geq s_{2kj}\;\mathrm{for}\;\mathrm{negative}\;\mathrm{index}\\ 1-2\left(x_{ikj}-s_{2kj}\right)/\left(s_{3kj}-s_{2kj}\right),\;\;s_{2kj}<x_{ikj}\leq s_{3kj}\;\mathrm{for}\;\mathrm{positive}\;\mathrm{index},\;\;s_{2kj}>x_{ikj}\geq s_{3kj}\;\mathrm{for}\;\mathrm{negative}\;\mathrm{index} \end{array}$$

(5) $$u_{3ikj}=\{\begin{array}{l} -1,\;\;s_{0kj}\leq x_{ikj}\leq s_{1kj}\;\mathrm{for}\;\mathrm{positive}\;\mathrm{index},\;\;s_{0kj}\geq x_{ikj}\geq s_{1kj}\;\mathrm{for}\;\mathrm{negative}\;\mathrm{index}\\ 1-2\left(s_{2kj}-x_{ikj}\right)/\left(s_{2kj}-s_{1kj}\right),\;\;s_{1kj}<x_{ikj}\leq s_{2kj}\;\mathrm{for}\;\mathrm{positive}\;\mathrm{index},\;\;s_{1kj}>x_{ikj}\geq s_{2kj}\;\mathrm{for}\;\mathrm{negative}\;\mathrm{index}\\ 1,\;\;s_{2kj}<x_{ikj}\leq s_{3kj}\;\mathrm{for}\;\mathrm{positive}\;\mathrm{index},\;\;s_{2kj}>x_{ikj}\geq s_{3kj}\;\mathrm{for}\;\mathrm{negative}\;\mathrm{index} \end{array}$$

where the larger the value of positive (negative) index, the higher (lower) the evaluation grade. *s*_1*kj*_ and *s*_2*kj*_ are the critical value of grade 1 and grade 2, and that of grade 2 and grade 3 for index *j* in subsystem *k*, respectively, while *s*_0*kj*_ and *s*_3*kj*_ are other critical values of grade 1 and grade 3, respectively.

For the components of ternary connection number in Formulas (3)–(5), i.e., *u_gikj_*, depending on whether the evaluation index sample value *x_ikj_* fell in the same, adjacent, or separated interval of evaluation grade *g*, and took the value of 1, within [−1, 1], or −1, respectively. Therefore, *u_gikj_* can be regarded as a relative difference degree function of variable fuzzy set for the proximity between evaluation index value and evaluation grade criteria. The corresponding relative membership degree is as follows [45]:

(6) $$a_{ikj}^{*}=0.5+0.5u_{1ikj},b_{ikj}^{*}=0.5+0.5u_{2ikj},c_{ikj}^{*}=0.5+0.5u_{3ikj},i=1,2,\ldots ,m;k=1,2,3,4;j=1,2,\ldots ,n_{k}$$

By the normalization of Formula (6), the components of ternary connection number for evaluation index of agricultural drought disaster risk can be finally obtained [33]:

(7) $$a_{ikj}=a_{ikj}^{*}/\left(a_{ikj}^{*}+b_{ikj}^{*}+c_{ikj}^{*}\right),\;\;b_{ikj}=b_{ikj}^{*}/\left(a_{ikj}^{*}+b_{ikj}^{*}+c_{ikj}^{*}\right),\;\;c_{ikj}=c_{ikj}^{*}/\left(a_{ikj}^{*}+b_{ikj}^{*}+c_{ikj}^{*}\right)$$

**Figure 2:**
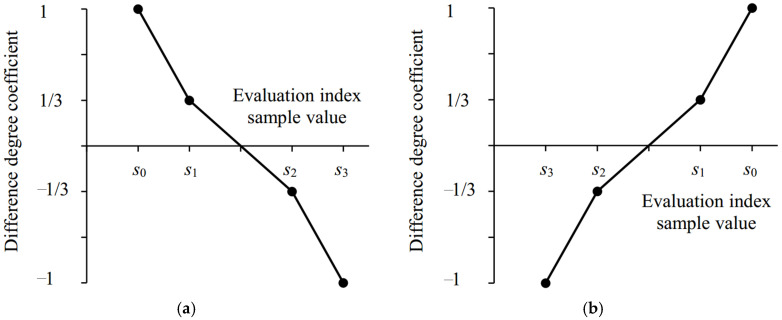
The value process of dynamic difference degree coefficient varying with evaluation index sample value for ternary connection number. (**a**) Positive index; (**b**) Negative index. The larger the value of positive (negative) index, the higher (lower) the evaluation grade.

(4) Difference degree coefficient *I* was the key to describing the uncertainty of set pair at a micro level [32,43], and an important source of this uncertainty was the sample data of research problem. In addition, the physical meaning of *I* can be interpreted as the quantity of the difference degree *b* transforming into similarity degree *a* or opposition degree *c*. The direction (to *a* or *c*) and the scale of transformation should be closely related to the proximity between the actual evaluation sample value and each evaluation grade. That was, the larger *a* (or *c*), the more *b* was transformed into *a* (or *c*). Therefore, *I* should vary with the evaluation sample [46]. In this study, the set pair composed of evaluation index sample value *x_ikj_* and evaluation grade criteria *s_gkj_* belonged to positive–negative relation; the value of *I* ranged within [−1, 1]. Moreover, when *I* ranged within [0, 1], *b* was transformed into *a*; when *I* ranged within [−1, 0], *b* was transformed into *c*. The absolute value of *I* represented the scale of transformation. Based on SPA and the above analysis, this study considered that the difference degree coefficient of ternary connection number for evaluation index of agricultural drought disaster risk, i.e., *I_ikj_* varied with the evaluation index sample value *x_ikj_*. The calculation method is shown in Figure 2 and Formula (8) [46]:

(8) $$I_{ikj}=\{\begin{array}{l} 1-\left(1-m\right)\left(x_{ikj}-s_{0kj}\right)/\left(s_{1kj}-s_{0kj}\right),\;\;s_{0kj}\leq x_{ij}\leq s_{1kj}\;\mathrm{for}\;\mathrm{positive}\;\mathrm{index},\;\;s_{0kj}\geq x_{ikj}\geq s_{1kj}\;\mathrm{for}\;\mathrm{negative}\;\mathrm{index}\\ -2m\left[x_{ikj}-\left(s_{1kj}+s_{2kj}\right)/2\right]/\left(s_{2kj}-s_{1kj}\right),\;\;s_{1kj}<x_{ikj}\leq s_{2kj}\;\mathrm{for}\;\mathrm{positive}\;\mathrm{index},\;\;s_{1kj}>x_{ikj}\geq s_{2kj}\;\mathrm{for}\;\mathrm{negative}\;\mathrm{index}\\ -1+\left(1-m\right)\left(s_{3kj}-x_{ikj}\right)/\left(s_{3kj}-s_{2kj}\right),\;\;s_{2kj}<x_{ikj}\leq s_{3kj}\;\mathrm{for}\;\mathrm{positive}\;\mathrm{index},\;\;s_{2kj}>x_{ikj}\geq s_{3kj}\;\mathrm{for}\;\mathrm{negative}\;\mathrm{index} \end{array}$$

(5) Substitute Formulas (3)–(8) into Formula (2) to calculate the ternary connection number of index *j* in subsystem *k* for evaluation sample *i*, i.e., *u_ikj_* [45]:

(9) $$u_{ikj}=a_{ikj}+b_{ikj}I_{ikj}+c_{ikj}J,\;\;i=1,2,\ldots ,m;k=1,2,3,4;j=1,2,\ldots ,n_{k}$$

According to the weight of index *j* in subsystem *k* obtained by Formula (1), i.e., *w_kj_*, calculate the ternary connection number of subsystem *k* for sample *i*, i.e., *u_ik_* [33]:

(10) $$u_{ik}=\sum_{j=1}^{n_{k}}w_{kj}a_{ikj}+\sum_{j=1}^{n_{k}}w_{kj}b_{ikj}I_{ikj}+\sum_{j=1}^{n_{k}}w_{kj}c_{ikj}J,\;\;i=1,2,\ldots ,m;k=1,2,3,4$$

Finally, the ternary connection number *u_i_* for evaluation sample *i* of agricultural drought disaster risk was obtained by the following formula [40]:

(11) $$u_{i}=\sum_{k=1}^{4}\sum_{j=1}^{n_{k}}w_{k}w_{kj}a_{ikj}+\sum_{k=1}^{4}\sum_{j=1}^{n_{k}}w_{k}w_{kj}b_{ikj}I_{ikj}+\sum_{k=1}^{4}\sum_{j=1}^{n_{k}}w_{k}w_{kj}c_{ikj}J,\;\;i=1,2,\ldots ,m$$

where *w_k_* is the weight of subsystem *k*, which can be determined by AGA-FAHP [45].

The connection number *u* calculated by Formulas (10) and (11) were used to evaluate the grade of agricultural drought disaster risk. It can be proved that *u* ∈ [−1, 1]. According to the grade corresponding to the critical value, *u* was divided into three grades, which corresponded to three statuses of agricultural drought disaster risk, respectively, i.e., low-risk *u* ∈ (0.667, 1.000], middle-risk *u* ∈ [−0.667, 0.667], and high-risk status *u* ∈ [−1.000, −0.667).

Meanwhile, to compare with the result of the connection number value, level eigenvalue method was used to calculate the grade value *h_i_* for evaluation sample *i* of agricultural drought disaster risk. Similarly, *h* was divided into three grades, which corresponded to low-risk *h* ∈ [1.0, 1.5), middle-risk *h* ∈ [1.5, 2.5], and high-risk status *h* ∈ (2.5, 3.0] [45]:

(12) $$h_{i}=\sum_{k=1}^{4}\sum_{j=1}^{n_{k}}w_{k}w_{kj}a_{ikj}+2\sum_{k=1}^{4}\sum_{j=1}^{n_{k}}w_{k}w_{kj}b_{ikj}+3\sum_{k=1}^{4}\sum_{j=1}^{n_{k}}w_{k}w_{kj}c_{ikj},\;\;i=1,2,\ldots ,m$$

In order to compare the information provided by connection number components and connection number value in agricultural drought disaster risk evaluation process, the difference degree *b* and difference degree item *bI* were respectively converted into a probability variable *p* based on information entropy theory [45,47], according to the following formula:

(13) $$p_{i}=\frac{{\left(\sum_{k=1}^{4}\sum_{j=1}^{n_{k}}w_{k}w_{kj}b_{ikj}\right)}^{2}}{\sum_{i=1}^{m}{\left(\sum_{k=1}^{4}\sum_{j=1}^{n_{k}}w_{k}w_{kj}b_{ikj}\right)}^{2}}\;\mathrm{and}\;p_{i}=\frac{{\left(\sum_{k=1}^{4}\sum_{j=1}^{n_{k}}w_{k}w_{kj}b_{ikj}I_{ikj}\right)}^{2}}{\sum_{i=1}^{m}{\left(\sum_{k=1}^{4}\sum_{j=1}^{n_{k}}w_{k}w_{kj}b_{ikj}I_{ikj}\right)}^{2}}$$

Then, the corresponding information entropy value *e* was obtained as follows [45,47]:

(14) $$e=-\frac{\sum_{i=1}^{m}p_{i}\ln p_{i}}{\ln m}$$

(6) The connection number *u_ikj_* of evaluation index calculated by Formula (9) was used to diagnose the main factors that made the high risk. It can be proved that *u_ikj_* ∈ [−1, 1]. According to the principle of equipartition, evaluation index was divided into five types, which were strong obstacle *u_ikj_* ∈ [−1.0, −0.6), middle obstacle *u_ikj_* ∈ [−0.6, −0.2), weak obstacle *u_ikj_* ∈ [−0.2, 0.2], weak promotion *u_ikj_* ∈ (0.2, 0.6], and strong promotion *u_ikj_* ∈ (0.6, 1.0]. Among them, strong obstacle index and middle obstacle index were the primary obstacle factors that seriously hindered the reduction of agricultural drought disaster risk, and meanwhile, the key objects of risk regulation and control [46].

Additionally, to compare with the diagnosis result of the connection number value, semipartial subtraction set pair potential *s*(*u*) corresponding to the ternary connection number of evaluation index was calculated to identify the obstacle factors of risk as follows [30]:

(15) $$s\left(u_{ikj}\right)=\left\{ \begin{array}{l} -1,\;\;\mathrm{when}\;a_{ikj}+b_{ikj}=0\\ 1,\;\;\mathrm{when}\;b_{ikj}+c_{ikj}=0\\ \left[a_{ikj}+b_{ikj}a_{ikj}/\left(a_{ikj}+b_{ikj}\right)\right]-\left[c_{ikj}+b_{ikj}c_{ikj}/\left(c_{ikj}+b_{ikj}\right)\right],\;\;\mathrm{when}\;a_{ikj}+b_{ikj}\neq 0\;\mathrm{and}\;b_{ikj}+c_{ikj}\neq 0 \end{array}\right.$$

It can be proved that *s*(*u_ikj_*) ∈ [−1, 1]. According to the principle of equipartition, *s*(*u_ikj_*) was divided into inverse potential *s*(*u_ikj_*) ∈ [−1.0, −0.6), partial inverse potential *s*(*u_ikj_*) ∈ [−0.6, −0.2), symmetrical potential *s*(*u_ikj_*) ∈ [−0.2, 0.2], partial identical potential *s*(*u_ikj_*) ∈ (0.2, 0.6], and identical potential *s*(*u_ikj_*) ∈ (0.6, 1.0], respectively. Among them, the index of *s*(*u_ikj_*) in inverse potential or partial inverse potential was the obstacle factors of agricultural drought disaster risk and should be paid more attention to in risk management [40].

### 2.2. Study Area

**Figure 3:**
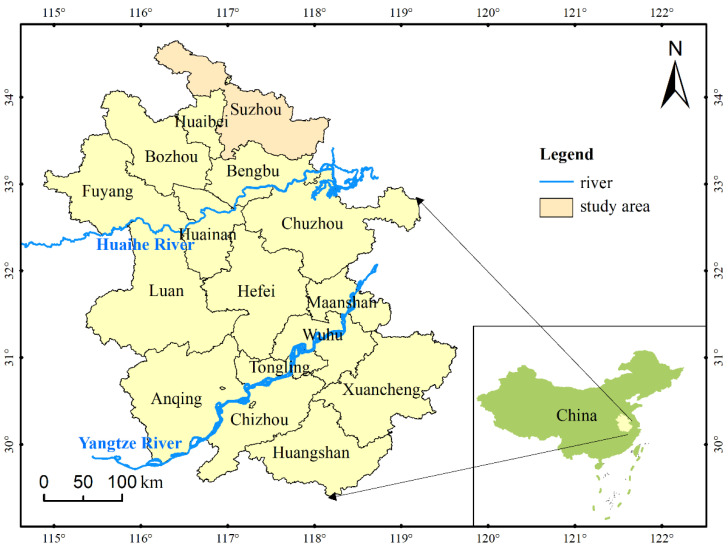
The location of Suzhou City in Anhui Province, China.

Suzhou City in Anhui Province was chosen as a typical grain production area for empirical study (Figure 3). Suzhou was located in the south of Huang-Huai Plain, the main production area of winter white and the important commodity grain export base in China [30]. However, due to its transition zone between temperate and subtropical monsoon climate, the distribution of annual precipitation was extremely uneven, and the precipitation in summer accounted for 50–60% of the annual precipitation [33]. Meanwhile, the monsoon had a significant impact on precipitation, resulting in a large inter-annual variation and frequent drought disaster [31]. In addition, the average sown area of crops in Suzhou from 2001 to 2020 was 9.99 × 10^5^ hectares, including 7.87 × 10^5^ hectares for grains, and the average grain yield was 3.63 × 10^9^ kg. However, frequent drought disasters have caused serious losses in agricultural production in Suzhou. For instance, the most severe autumn-winter drought disaster in recent ten years continued from October 2008 to February 2009. According to the statistics, the total affected area of wheat by droughts in the city was 3.50 × 10^5^ hectares, accounting for 91.7% of the sown area of wheat, and the seriously affected area was 1.76 × 10^5^ hectares [48]. Furthermore, two-phased drought disasters occurred in 2012. The affected area by the first was 3.99 × 10^5^ hectares, and that of the second was 2.60 × 10^5^ hectares, causing a direct economic loss of about RMB 426 million. A heavy drought disaster happened from the last ten days of July to the middle ten days of August in 2013. The largest affected area was 1.76 × 10^5^ hectares, including 0.4 × 10^5^ hectares seriously affected [49]. Suzhou was a traditional agriculture-oriented city, and agricultural production was an important source of farmers’ income and local economic development. Therefore, conducting the evaluation and diagnosis of agricultural drought disaster risk in Suzhou was of great significance for ensuring stable social economy and food security.

### 2.3. Data Acquisition

The sample data used for evaluation and obstacle factor diagnosis of agricultural drought disaster risk in Suzhou City were obtained from Water Resources Bulletin of Anhui Province (2007–2017), Statistical Yearbook of Anhui Province (2008–2018), Statistical Yearbook of Suzhou City (2008–2018), Water Conservancy Yearbook of Anhui Province (2008–2018), and Drought Resistance Planning in Anhui Province.

The hydro-meteorological dataset used in this study included precipitation, evaporation, and soil moisture data. The precipitation data (2007–2017) were from the Chinese national meteorological Suzhou station, which can be accessed from The China Meteorological Data Service Center (http://data.cma.cn (accessed on 6 February 2022)). The evaporation data (2007–2017) were calculated by FAO-56 Penman-Monteith method [50] according to daily maximum and minimum air temperature, relative humidity, wind speed, vapor pressure, and duration of sunshine, which were from the Chinese national meteorological Suzhou station, they can be accessed from http://data.cma.cn (accessed on 6 February 2022). The soil moisture data (2007–2017) were from the Chinese national soil moisture monitoring Suzhou station, and the soil moisture report in Anhui Province can be accessed from http://slt.ah.gov.cn/tsdw/swj/sqtx/index.html (accessed on 14 February 2022).

## 3. Results and Discussion

### 3.1. Evaluation System and Weights of Agricultural Drought Disaster Risk

According to the evaluation target and construction principle of the evaluation system for agricultural drought disaster risk, based on the system structure of drought disaster risk [17,31], the risk system was divided into four subsystems, i.e., drought hazard, drought damage sensitivity, exposure to drought, and drought resistance. By the analysis of characteristics for these four subsystems, and meanwhile referring to related research [25,30,33,36,44] and the actual situation of water resources, social economy, agricultural production, and water conservancy projects in the study area, an evaluation system of agricultural drought disaster risk (including 4 subsystems, in total 21 indexes, i.e., *X*_1_–*X*_21_) and the corresponding evaluation grade criteria (low, middle, and high risk) were established, as shown in Table 1. Furthermore, AGA-FAHP [45] and related research [30,44] were used to determine the weight of each subsystem and evaluation index (Table 1).

### 3.2. Evaluation of Agricultural Drought Disaster Risk in Suzhou from 2007 to 2017

**Figure 4:**
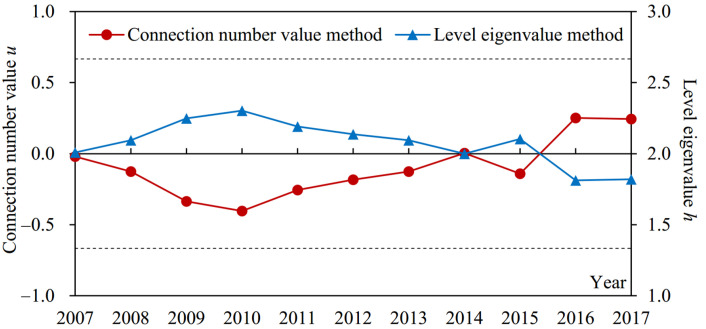
The evaluation grades of agricultural drought disaster risk in Suzhou from 2007 to 2017 obtained by two methods. The two dashed lines from top to bottom represent the boundaries of the connection number value for low risk and middle risk status (0.667), and that for middle risk and high risk status (−0.667), respectively.

**Figure 5:**
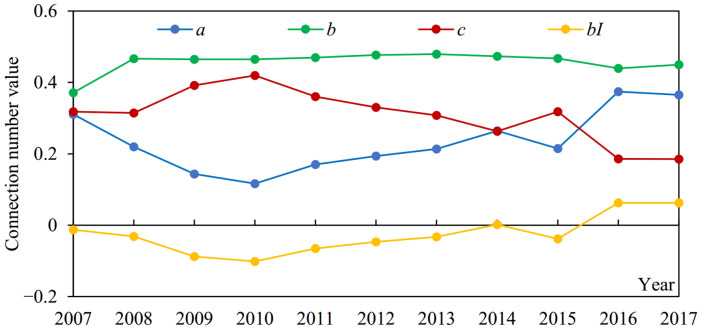
The components of the connection number for agricultural drought disaster risk evaluation in Suzhou from 2007 to 2017.

**Figure 6:**
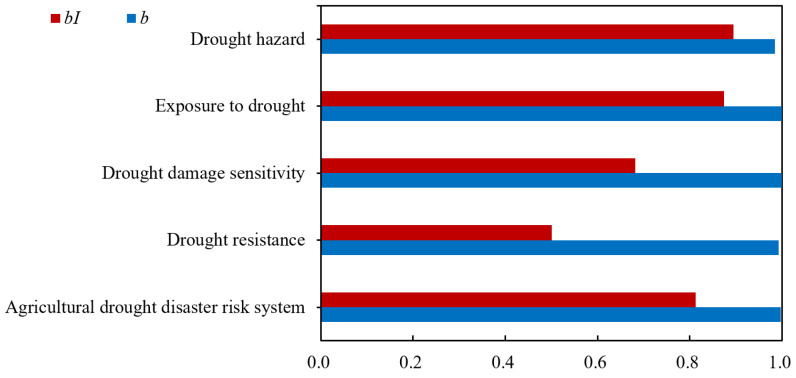
The information entropy values of the difference degree *b* and difference degree item *bI* for agricultural drought disaster risk system and its four subsystems in Suzhou.

**Figure 7:**
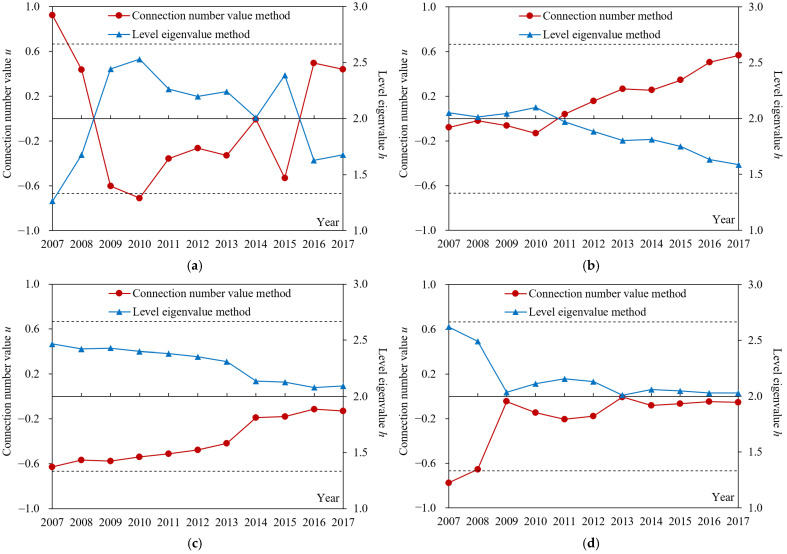
The evaluation grades of agricultural drought disaster risk subsystems in Suzhou from 2007 to 2017 obtained by two methods. (**a**) Drought hazard subsystem; (**b**) Drought damage sensitivity subsystem; (**c**) Exposure to drought subsystem; (**d**) Drought resistance subsystem. The two dotted lines from top to bottom represent the boundaries of the connection number value for low risk and middle risk status (0.667), and that for middle risk and high risk status (−0.667), respectively.

**Figure 8:**
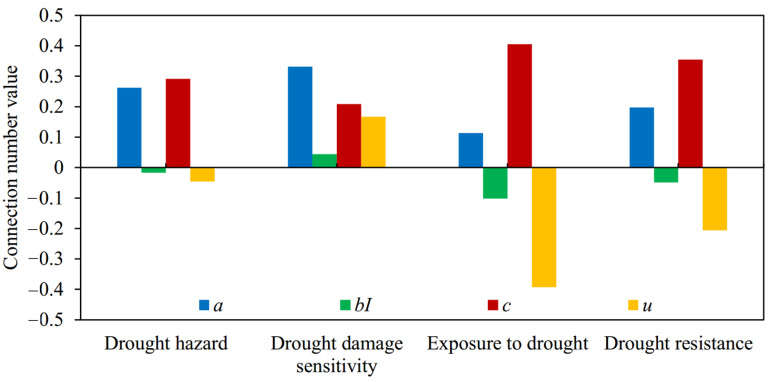
The average values of the connection number and its components for agricultural drought disaster risk subsystem evaluation in Suzhou from 2007 to 2017.

**Figure 9:**
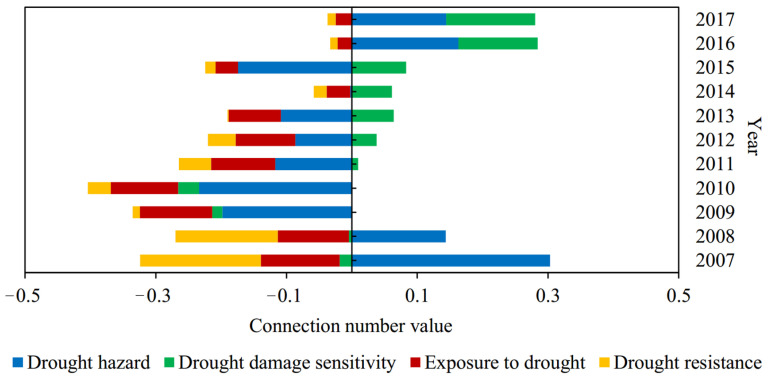
The connection number values of agricultural drought disaster risk subsystem evaluation in Suzhou from 2007 to 2017.

The index sample data in Suzhou from 2007 to 2017 and the corresponding grade criteria shown in Table 1 were substituted into Formulas (3)–(7) to obtain the components of the connection number for each index. The dynamic difference degree coefficient of the connection number was calculated according to Figure 2 and Formula (8). Then, the weights of subsystem and index in Table 1 and Formulas (9)–(11) were adopted to calculate the connection number values of index, subsystem, and drought disaster risk system, respectively. Meanwhile, Formula (12) was used to calculate the grade obtained by the level eigenvalue method. Furthermore, although the results of the connection number value and level eigenvalue methods both reflect the evaluation grade, the larger the former, the lower the risk grade, while the larger the latter, the higher the risk grade. The evaluation results of agricultural drought disaster risk in Suzhou are shown in Figure 4, Figure 5, Figure 6, Figure 7, Figure 8 and Figure 9.

(1) From the results obtained by two methods (Figure 4), the variation trends of evaluation grade for agricultural drought disaster risk in Suzhou from 2007 to 2017 calculated by connection number value and level eigenvalue were consistent. Nevertheless, compared with the level eigenvalue, the variation amplitude year by year of the connection number value was more obvious, and the difference among evaluation grades was more significant. For example, the difference between the maximum and minimum of the connection number value relative to its value range ((0.251 − (−0.404))/(1 − (−1)) × 100% = 32.75%)) was higher than that of level eigenvalue ((2.303 − 1.812)/(3 − 1) × 100% = 24.55%)). Moreover, the connection number value in 2016 changed greatly; the difference with 2015 was 0.392, which was higher than that of the level eigenvalue (0.292). It indicated that the evaluation method of agricultural drought disaster risk in this study could take full advantage of the information from samples. The dynamic difference degree coefficient varying with the actual evaluation sample can further quantify the uncertainty between evaluation index value and evaluation grade criteria and determine the complete connection number value. Therefore, the evaluation results were reasonable, reliable, and had a higher sensitivity.

According to the connection number value of agricultural drought disaster risk evaluation in Suzhou from 2007 to 2017 (Figure 4), it decreased from −0.201 in 2007 to −0.404 in 2010 and then increased to 0.243 in 2017. Among them, a fluctuation was found in 2015, and the connection number value decreased. The average connection number values during 2007–2010 and 2011–2017 were −0.222 and −0.030, respectively, and the connection number values in 2016 and 2017 were larger than 0.2 (Figure 4). It reflected that the agricultural drought disaster risk in Suzhou from 2010 to 2017 was always in middle-risk status (−0.667 ≤ *u* ≤ 0.667). Nevertheless, the risk presented a decreasing trend from 2010, especially since 2016, it had gradually developed to low-risk status. Therefore, in recent years, the agricultural drought disaster risk in Suzhou was still quite severe, but it tended to be improved. The drought disaster risk management may not arouse enough attention before 2010, so it was necessary to strengthen scientific and reasonable risk control measures. These results were consistent with the studies of Wang et al. [44] and Jin et al. [30].

(2) According to the components of the connection number for agricultural drought disaster risk evaluation in Suzhou during 2007–2017 (Figure 5), the similarity degree *a* first decreased and then increased, while the opposition degree *c* first increased and then decreased (with 2010 as the boundary), and the difference degree *b* basically did not change. Moreover, the difference degree item *bI* presented the same trend as that of *a*, but the amplitude was smaller. It can be seen that the change trends of *a* and *bI* were consistent with that of *u*, while the trend of *c* was contrary to that of *u*. These explained the reason why *u* first decreased and then increased in Figure 4 combined with Formula (11). Additionally, *c* was overall larger than *a*, but the difference decreased gradually; *a* had exceeded *c* in 2016 and 2017 (Figure 5). *bI* was always smaller than *a*. The average values of *a*, *bI*, and *c* during 2007–2017 were 0.235, −0.026, and 0.308, respectively. These findings accorded with the above results that the situation of agricultural drought disaster risk in Suzhou was still serious, but it presented a trend of improvement (Figure 4).

The change of *b* was small from 2007 to 2017; the standard deviation was 0.031, but the change of *bI* was relatively significant; the standard deviation was 0.053 (Figure 5). In addition, according to Formulas (13) and (14), the information entropy values of *bI* were all lower than those of *b* for the agricultural drought disaster risk system and its four subsystems, especially for the drought resistance subsystem (Figure 6). The information entropy values of *b* and *bI* were 0.992 and 0.501 for drought resistance, respectively (Figure 6). Based on information entropy theory [45,47], it reflected that *bI* provided more information in the risk evaluation process. Furthermore, according to Formulas (11) and (12), compared with the level eigenvalue method, which only took *a*, *b*, and *c* into account, the connection number value method used in this study additionally considered the effect of the difference degree coefficient *I*, which varied dynamically with the evaluation sample value and utilized more information, bringing more accurate and reliable evaluation results. It explained why the above risk evaluation results of the connection number value had a higher sensitivity (Figure 4). Overall, the agricultural drought disaster risk in Suzhou showed a decreasing trend, but the situation was still severe. Therefore, it was necessary to analyze the risk driving mechanism from subsystem and index aspects, identify key factors hindering the reduction of risk, and formulate targeted regulation measures.

### 3.3. Evaluation of Agricultural Drought Disaster Risk Subsystems in Suzhou from 2007 to 2017

In order to explain the causes of agricultural drought disaster risk in Suzhou, this study further revealed the key driving elements and obstacle factors of risk from four subsystems.

(1) According to the evaluation results of agricultural drought disaster risk subsystems in Suzhou during 2007–2017 obtained by two methods (Figure 7), the changes in the evaluation grade of four subsystems calculated by connection number value and level eigenvalue methods were consistent, and the difference among evaluation grades calculated by connection number value was more significant. For instance, the difference between the maximum and minimum connection number value for drought damage sensitivity relative to its value range ((0.565 − (−0.132))/(1 − (−1)) × 100% = 34.85%) was larger than that of the level eigenvalue ((2.100 − 1.587)/(3 − 1) × 100% = 25.65%). It indicated that the evaluation results of the risk subsystem were reasonable, reliable, and with higher sensitivity.

The connection number value of drought hazard during 2007–2017 changed greatly. It first decreased and then increased. The connection number values of drought damage sensitivity, exposure to drought, and drought resistance gradually increased, with a small variation amplitude. However, four subsystems were still in middle-risk status (Figure 7). It showed that in recent years, the drought hazard, drought damage sensitivity, and exposure to drought in Suzhou had reduced, and the drought resistance had increased, but their overall situations were still serious, which were consistent with the above connection number values of risk evaluation (Figure 4). In addition, the change in connection number value of each subsystem accorded with its attribute and role in the drought disaster risk system [25]. Drought hazards reflected the natural attributes influencing drought in a study region, including precipitation, evaporation, water resources, etc.; they had strong random uncertainty and thus had a large variation amplitude (Figure 7a) [7,9]. The other three subsystems showed the drought resistance capacity and adaptability of the drought-affected body and reflected the social attributes influencing drought. With the development of economy, science and technology, and the layout of water conservancy projects, the status of these three subsystems has been improved steadily (Figure 7b–d)) [36]. It revealed that the subsystems of agricultural drought disaster risk and their indexes chosen in this study were reasonable and effective and with physical mechanisms.

(2) According to the average values of the connection number and their components for agricultural drought disaster risk evaluation in Suzhou during 2007–2017 (Figure 8), the magnitude sorting of *bI* among four subsystems was fully consistent with *u*, which accorded with the above physical meaning of the difference degree coefficient analyzed in this study. That was, when *a* > *c*, *b* was transformed into a, *I* > 0, when *a* < *c*, *b* was transformed into *c*, *I* < 0, and when *a* = *c*, *I* = 0. It showed that the calculation method of dynamic difference degree coefficient varying with the evaluation sample was reasonable and reliable and completely reflected its role and function in the structure of the connection number. Furthermore, the average connection number values of drought hazard, drought damage sensitivity, exposure to drought, and drought resistance were −0.046, 0.167, −0.393, and −0.206, respectively. Therefore, from the perspective of the multi-year average level, the risk status of drought damage sensitivity was optimal, while those of exposure to drought and drought resistance were relatively worse. Combined with Figure 7, the connection number values of exposure to drought and drought resistance were negative in recent years, while those of drought damage sensitivity were basically positive and even gradually improved from middle risk to low risk. Therefore, exposure to drought and drought resistance were the main factors that hindered the reduction of agricultural drought disaster risk in Suzhou and should arouse attention. It was crucial to implement effective measures for reducing the degree of exposure to drought and strengthening drought resistance capacity.

According to the connection number values of agricultural drought disaster risk subsystem evaluation in Suzhou during 2007–2017 (Figure 9), the connection number value of drought hazard first decreased and then increased and became positive since 2016; its absolute value was always relatively large. The connection number value of drought damage sensitivity changed from negative to positive in 2011 and then increased significantly. Nevertheless, those of exposure to drought and drought resistance also increased constantly, but the amplitudes were small, and the values were always negative. It revealed that the drought damage sensitivity and drought hazard in Suzhou during recent years reduced significantly, which were the main reason for the improvement of risk status above (Figure 4). Meanwhile, the exposure to drought and drought resistance had been improved slightly, which had little impact on risk status. The primary subsystems which affected the risk in Suzhou had gradually changed from drought hazard, exposure to drought, and drought resistance in the past, to drought hazard and drought damage sensitivity at present. In addition, in the past few years, the connection number values of four subsystems had not exceeded 0.2, and most of them were negative. Especially the high exposure to drought and the weak drought resistance capacity had become the main factors that seriously impeded the reduction of risk (Figure 4). To sum up, due to the strong random uncertainty of drought hazard [51], this factor cannot be controlled and predicted easily [31]. Therefore, the key to controlling agricultural drought disaster risk in Suzhou was to steady the level of drought damage sensitivity for drought-affected bodies; the difficulties were to reduce exposure to drought and improve resistance capacity.

### 3.4. Obstacle Factor Diagnosis of Agricultural Drought Disaster Risk in Suzhou from 2007 to 2017

The variations in the status of the agricultural drought disaster risk system and its subsystems have been analyzed above. Then, the connection number value and semipartial subtraction set pair potential of evaluation index were used to diagnose the obstacle factors of risk so as to provide a basis for drought disaster risk regulation in Suzhou.

(1) The components of the connection number and the difference degree coefficient of the evaluation index were used to calculate the connection number value and semipartial subtraction set pair potential by Formulas (9) and (15), respectively, as shown in Table 2. It can be seen that, from the multi-year average aspect, the number of indexes that belonged to middle or strong obstacle (−1.0 ≤ *u* < −0.2) were 1, 1, 3, and 4 in drought hazard, drought damage sensitivity, exposure to drought, and drought resistance subsystems, respectively, and those which belonged to weak or strong promotion (0.2 < *u* ≤ 1.0) were 1, 2, 0, and 2, respectively. These were consistent with the above result that the overall status of drought damage sensitivity was optimal, while those of exposure to drought and drought resistance were relatively worse (Figure 8). It reflected that the number of indexes that belonged to the strong promotion and strong obstacle in a risk subsystem could approximately judge the risk status of such subsystem. That was, the less strong promotion indexes and the more strong obstacle indexes, the worse the risk status, and vice versa. These basically accorded with the results of semipartial subtraction set pair potential, but there were some differences (Table 2). For example, the average connection number value of annual precipitation *X*_2_ was −0.150 (weak obstacle), while that of semipartial subtraction set pair potential was −0.247 (partial inverse potential). The average connection number value of the percentage of cultivated land *X*_12_ was −0.597 (middle obstacle), while that of semipartial subtraction set pair potential was −0.608 (inverse potential). It can be found that the value of semipartial subtraction set pair potential was relatively small.

From the perspective of multi-year average level, the percentage of agricultural population *X*_7_, population density *X*_11_, and percentage of effective irrigation area *X*_18_ were strong obstacle indexes. In addition, the water resources amount per unit area *X*_4_, percentage of cultivated land *X*_12_, multiple cropping index *X*_13_, percentage of reservoir regulation and storage *X*_16_, monitoring and warning capacity *X*_20_, and percentage of water-saving irrigation area *X*_21_ were middle obstacle indexes (Table 2). Therefore, these indexes were the main factors that severely hindered the effective reduction of agricultural drought disaster risk, also the key and difficult objects of risk regulation and control in Suzhou.

**Figure 10:**
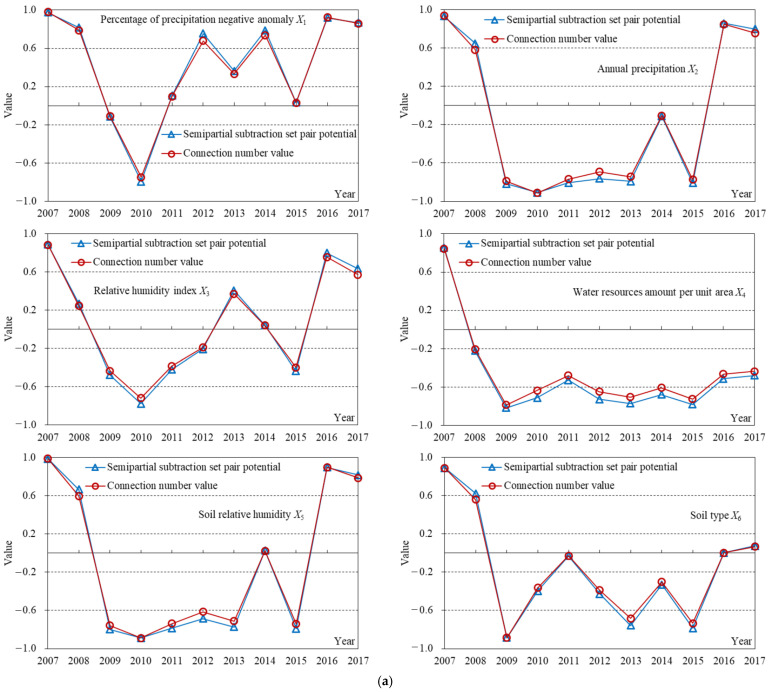

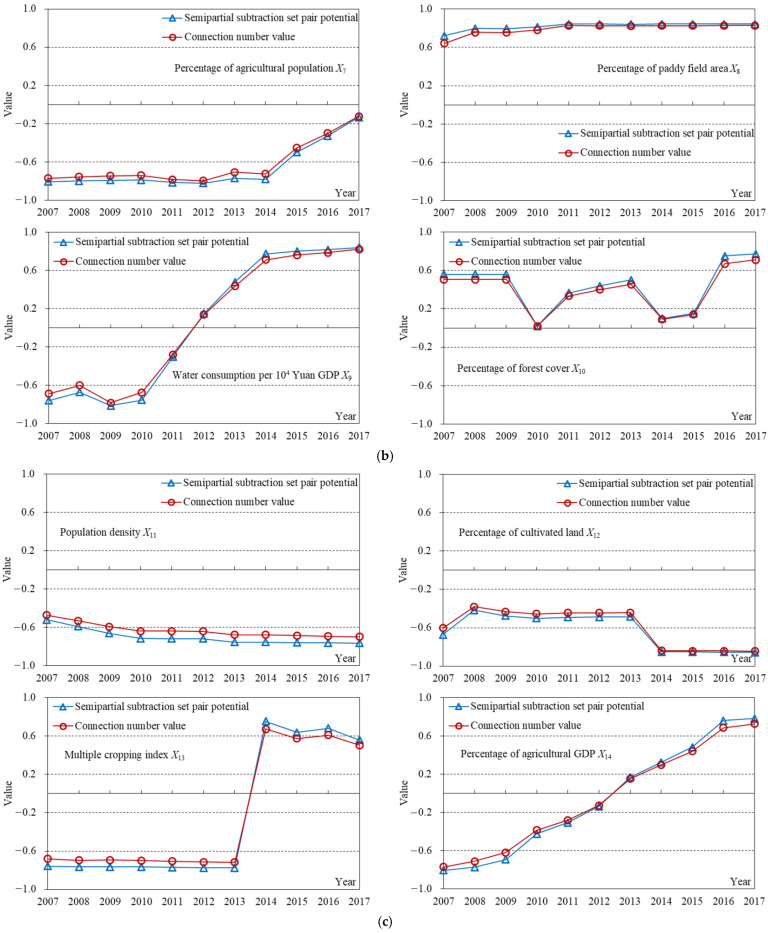

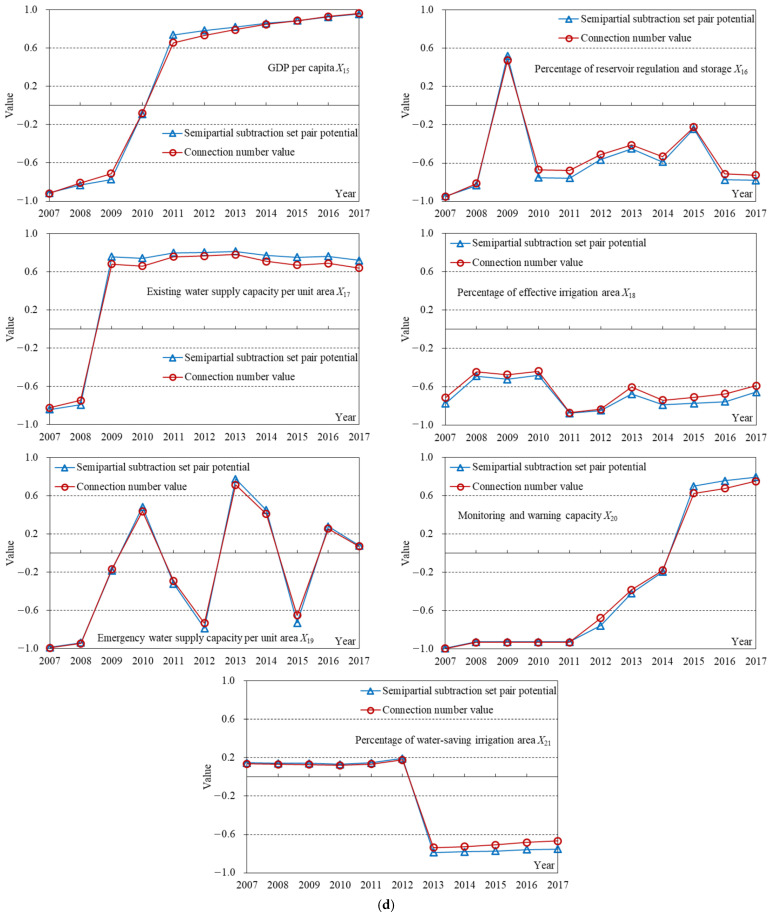
The connection number values of agricultural drought disaster risk evaluation index in Suzhou from 2007 to 2017. (**a**) Drought hazard subsystem; (**b**) Drought damage sensitivity subsystem; (**c**) Exposure to drought subsystem; (**d**) Drought resistance subsystem. The four dotted lines from top to bottom represent the boundaries of the connection number value for strong promotion and weak promotion types (0.6), and those for weak promotion and weak obstacle (0.2), weak obstacle and middle obstacle (−0.2), middle obstacle and strong obstacle types (−0.6), respectively.

(2) The connection number values and semipartial subtraction set pair potential of 21 indexes during 2007–2017 were shown in Figure 10. The change in connection number value of each index was analyzed, the key driving factors of risk were identified from the index level. Those indexes which had a small fluctuation of the connection number value and always belonged to strong or middle obstacles were the primary reasons for high risk. They can be diagnosed as the obstacle factors of risk and the crucial objects of risk control [46].

The variation trends of the connection number value for the evaluation index during 2010–2017 were consistent with those of semipartial subtraction set pair potential, whose overall variation amplitude was not significant. Nevertheless, when the change between two years was big, the amplitude of semipartial subtraction set pair potential was larger (Figure 10). For instance, the connection number value of annual precipitation *X*_2_ in 2007 and 2008 were 0.939 and 0.583 (with a difference of 0.356), respectively, while the semipartial subtraction set pair potential were 0.933 and 0.649 (0.284), respectively. The connection number value of the percentage of agricultural population *X*_7_ in 2012 and 2013 were −0.795 and −0.706 (with a difference of 0.089), respectively, while the semipartial subtraction set pair potential were −0.822 and −0.770 (0.052), respectively. However, the connection number value of multiple cropping index *X*_13_ in 2013 and 2014 were −0.717 and 0.676 (with a difference of 1.393), respectively, but the semipartial subtraction set pair potential were −0.776 and 0.755 (1.531), respectively. The connection number value of the percentage of reservoir regulation and storage *X*_16_ in 2009 and 2010 were 0.472 and −0.669 (with a difference of 1.141), but the semipartial subtraction set pair potential were 0.521 and −0.751 (1.272), respectively (Figure 10). It indicated that the connection number value calculated in this study was reasonable and reliable, which can identify the status change of the agricultural drought disaster risk evaluation index more accurately and sensitively.

As for the drought hazard subsystem, the obstacle factors of agricultural drought disaster risk, which always belonged to middle or strong obstacle indexes (the connection number value of index *u* < −0.2), included *X*_2_, *X*_4_, *X*_5_, and *X*_6_ (Figure 10a). It reflected that this subsystem was generally in middle-risk status during recent years (Figure 7a), which was due to less amount of precipitation and water resources, low soil moisture content, and poor soil water retention. Additionally, the connection number value of the index was used to analyze the reasons for the change in subsystem status, which provided guidance for risk control. Drought hazard decreased during 2010–2014 (Figure 7a) mainly because the percentage of precipitation negative anomaly *X*_1_ gradually developed from a strong obstacle in 2010 (−0.748) to strong promotion in 2014 (0.737) (Figure 10a). Drought hazard suddenly increased in 2015 (Figure 7a), mainly due to the fact that *X*_1_ drew back to weak obstacles again (0.031). Meanwhile, the annual precipitation *X*_2_ and soil relative humidity *X*_5_ deteriorated from a weak obstacle in 2014 to a strong obstacle in 2015 (Figure 10a). Moreover, drought hazards were reduced in 2016 because all indexes in this subsystem had been improved (Figure 7a and Figure 10a). Therefore, precipitation was the main factor that influenced the drought hazard in Suzhou, but it had strong random uncertainty and large fluctuation [51]. In addition, Suzhou was located in the transition zone between temperate and subtropical monsoon climate, characterized by an uneven distribution of annual precipitation, a large difference in inter-annual precipitation, and frequent droughts [33]. For example, from October 2008 to February 2009, the average precipitation in Suzhou was only about 20 mm, accounting for less than 20% of annual precipitation, causing the most serious autumn-winter drought in the past 10 years [48]. Furthermore, from the last ten days of July 2013 to the middle ten days of August 2013, the precipitation was less than the multi-year average value, and the temperature above 35 °C exceeded 27 days, resulting in severe drought [49]. These actual situations can be fully reflected by the obvious deterioration of the status of the drought hazard subsystem (Figure 7a) and its indexes (Figure 10a).

Some studies showed that due to the intensification of global climate change, such as a significant decrease in precipitation but a simultaneous increase in temperature [52], the frequency, intensity, and scope of influence of drought were increasing [53,54]. For instance, the southwest China extreme drought in 2010 [55], the 2010–2013 southern United States drought [56], and the severe drought problems in the Mediterranean area [57,58]. Therefore, climate change might be a main driving force that affects the hydrological cycle [52]. However, Koutsoyiannis [59] made an innovative discovery that although there seemed to be an increase in temperature in the last decades, the hydrological cycle did not seem to intensify from a global-scale analysis of water balance using gridded data. The hydrological cycle is an extremely complex process which influenced by lots of factors. The relationship between climate change and droughts should be further studied by a long series of observational data and coupled with accurate global climate model simulations.

In recent decades, drought has occurred frequently, and the influences have increased remarkably [53,54]. Nevertheless, some research indicated that the droughts were not very rare if one focused beyond the last 50 years on the climate dynamics of the past thousands of years. In fact, H.E. Hurst was one of the first that identified 70 years ago that the droughts might be explained by the so-called Hurst phenomenon [60,61,62,63,64]. Since then, this phenomenon has been identified in numerous key hydrological cycle processes [65]. Therefore, the actual drought hazard and drought disaster risk can be further objectively assessed by considering the periodicity of drought in a long series of climate change data.

The obstacle factor in the drought damage sensitivity subsystem was *X*_7_ (Figure 10b). It showed that the transformation of this subsystem from middle risk to low risk in recent years (Figure 7b) was impeded by a large percentage of the agricultural population. This problem was not mitigated until 2015; *X*_7_ developed into a weak obstacle in 2017 (−0.123) from a strong obstacle in 2014 (−0.724). Suzhou was a city depending on agricultural production, with a significant influence of drought on agriculture and strong drought damage sensitivity. With the urbanization and industrial upgrading, the percentage of agriculture was decreasing continuously, and the drought damage sensitivity of economic and social drought-affected bodies reduced. Drought damage sensitivity during 2010–2017 reduced significantly (Figure 7b) mainly because the water consumption per RMB10^4^ GDP *X*_9_ was gradually improved from a strong obstacle in 2010 (−0.675) to strong promotion in 2017 (0.820) (Figure 10b). It indicated that with the development of the economy, science and technology, and government guidance, social water-saving awareness and industrial water-saving technology were enhanced, and water utilization efficiency significantly raised, which effectively reduced the drought damage sensitivity. In addition, the reason why this subsystem was in a better risk status for a long time (Figure 7b) was that the percentage of paddy field area *X*_8_ was always strong promotion; meanwhile, the percentage of forest cover *X*_10_ fluctuated around weak promotion (Figure 10b). Therefore, planting drought tolerant crops such as wheat and maize [34] and maintaining a high forest cover [30] were the key to ensuring low drought damage sensitivity in Suzhou.

The obstacle factors in exposure to the drought subsystem included *X*_11_ and *X*_12_ (Figure 10c). It reflected that the connection number value of this subsystem was always lower than 0 and in middle-risk status (Figure 7c) because of a high population density and a large cultivated land area. When a drought occurred, for the region with a higher population density and a larger cultivated land area, there were more drought-affected bodies exposed to drought, which accorded with the connotation of exposure [19,25]. Furthermore, exposure decreased significantly during 2013–2017 (Figure 7c) because multiple cropping index *X*_13_ suddenly developed from a strong obstacle in 2013 (−0.717) to strong and weak promotion in 2014 and later, meanwhile, the percentage of agricultural GDP *X*_14_ gradually developed from weak obstacle to strong promotion (Figure 10c). Therefore, optimization of planting structure, appropriate development of agricultural production, improvement of production efficiency, and reasonable scale and layout of urban-rural development were the crucial elements to lower the exposure degree in Suzhou.

The obstacle factors in the drought resistance subsystem included *X*, *X*_18_, and *X*_21_ (Figure 10d). It showed that the improvement of this subsystem status was seriously hindered, and the connection number value during 2009–2017 fluctuated around 0 (Figure 7d) because of limited reservoir regulation and storage capacity, small effective irrigation area, and water-saving irrigation area. Drought resistance presented significant enhancement during 2008–2009 (Figure 7d) mainly due to the fact that the existing water supply capacity per unit area *X*_17_ was improved from a strong obstacle in 2008 (−0.747) to strong promotion in 2009 (0.681); meanwhile, *X*_16_ developed from strong obstacle (−0.813) to weak promotion (0.472) (Figure 10d). Moreover, the continuous increase in per capita GDP, existing water supply capacity, and monitoring and warning capacity strongly guaranteed the drought resistance capacity (Figure 10d). From the perspective of drought disaster risk system structure, the improvement of drought resistance capacity was the most effective and workable means for reducing risk [38]. For this reason, the keys to reducing agricultural drought disaster risk in Suzhou were to implement reservoir dredging and reconstruction, improve water conservancy infrastructure systems such as farm irrigation, lift water-saving irrigation technology, promote social and economic development, and ensure existing water supply capacity and drought warning capacity. These results were in line with the actual drought resistance situations in Suzhou. For instance, 652,000 people, 46,000 electro-mechanical wells, 59 pump stations, and RMB 100.79 million funds were provided for drought resistance in 2009 [50]. Additionally, 57,000 people, 13,000 wells, 16 pump stations, and RMB 52.67 million funds were used for drought resistance in 2013 [51]. These facts were consistent with the results that the drought hazard (Figure 7a) and drought resistance capacity (Figure 7d) both significantly increased in 2009 and 2013.

The calculation method of dynamic difference degree coefficient in this study is only applicable for ternary connection numbers. For the research problems with more than three evaluation grades, such as four-element and five-element connection numbers, the formula of dynamic difference degree coefficient should be deduced again. In addition, the relationship between evaluation index value and difference degree coefficient in Figure 2 and Formula (8) may not always be linear; the result of the nonlinear function could be compared.

The critical values of the grade for the evaluation index in Table 1 are determined and verified according to the actual agricultural drought disaster situation in Suzhou City, and they could not be directly used in other areas. The critical values have a great impact on evaluation results, while they may vary with various natural and social factors affecting drought disaster risk. Therefore, reasonably determining the critical values of grades is an important work for future drought disaster risk evaluation and diagnosis research.

## 4. Conclusions

In order to deal with the uncertainty between the evaluation sample and evaluation criteria, a calculation method of dynamic difference degree coefficient was adopted to fully utilize the evaluation sample information. Furthermore, information entropy values of the difference degree and difference degree item were respectively calculated to compare the information provided by connection number components and connection number value in the evaluation process. Then a quantitative evaluation and obstacle factor diagnosis model of agricultural drought disaster risk was established, and an empirical study was carried out in Suzhou City of Anhui Province, China. The conclusions are shown as follows:

(1) The agricultural drought disaster risks in Suzhou during 2010–2017 were all in middle-risk status. Furthermore, the risk presented a decreasing trend from 2010. Especially since 2016, the risk status had been improved significantly and gradually developed to low risk. It is necessary to continue strengthening scientific measures of risk control. In addition, the information entropy values of the difference degree item *bI* were all lower than those of the difference degree *b* for the agricultural drought disaster risk system and its four subsystems, reflecting that *bI* provided more information in the risk evaluation process.
(2) The drought damage sensitivity and drought hazard in Suzhou reduced markedly during recent years, which mainly accounted for the improvement of agricultural drought disaster risk status. However, high exposure to drought and weak drought resistance capacity were important factors that hindered the effective reduction of risk. The key to regulating risk was to maintain the level of drought damage sensitivity, and the difficulties were to reduce exposure to drought and improve drought resistance capacity.
(3) The percentage of the agricultural population, population density, and percentage of effective irrigation area were strong obstacle indexes. In addition, the water resources amount per unit area, percentage of cultivated land area, multiple cropping index, percentage of reservoir regulation and storage, monitoring and warning capacity, and percentage of water-saving irrigation were middle obstacle indexes. These were the main obstacle factors of agricultural drought disaster risk in Suzhou, and also the key points and difficulties of risk regulation and control, which should be paid more attention to.
(4) The evaluation and obstacle factor diagnosis results of agricultural drought disaster risk in Suzhou City in accord with the actual situation. The evaluation and diagnosis method based on connection number and information entropy is reasonable and reliable. It provides an effective approach to identifying the level of agricultural drought disaster risk and its key obstacle factors in important grain production areas and also supports the decisions of regional drought disaster risk prevention and control.

## Figures and Tables

**Table 1:** Evaluation system and grade criteria of agricultural drought disaster risk [30,44].

Evaluation System	Evaluation Subsystem	Evaluation Index	Evaluation Grade Criteria			Subsystem Weight	Index Weight	Index Type *
			Grade 1 (Low Risk)	Grade 2 (Middle Risk)	Grade 3 (High Risk)			
agricultural drought disaster risk system	drought hazard subsystem	percentage of precipitation negative anomaly *X*_1_ (%)	≤10	(10, 20]	>20	0.329	0.069	positive
		annual precipitation *X*_2_ (mm)	≥900	[800, 900)	<800		0.057	negative
		relative humidity index *X*_3_ (%)	≥−0.40	[−0.60, −0.40)	<−0.60		0.049	negative
		water resources amount per unit area *X*_4_ (10^4^ m^3^ km^−2^)	≥45	[25, 45)	<25		0.057	negative
		soil relative humidity *X*_5_ (%)	≥75	[65, 75)	<65		0.044	negative
		soil type *X*_6_	≥0.70	[0.50, 0.70)	<0.50		0.052	negative
	drought damage sensitivity subsystem	percentage of agricultural population *X*_7_ (%)	≤70	(70, 85]	>85	0.241	0.060	positive
		percentage of paddy field area *X*_8_ (%)	≤2	(2, 10]	>10		0.068	positive
		water consumption per RMB10^4^ GDP *X*_9_ (m^3^ per RMB10^4^)	≤100	(100, 150]	>150		0.062	positive
		percentage of forest cover *X*_10_ (%)	≥30	[20, 30)	<20		0.052	negative
	exposure to drought subsystem	population density *X*_11_ (person km^−2^)	≤450	(450, 650]	>650	0.192	0.047	positive
		percentage of cultivated land *X*_12_ (%)	≤40	(40, 50]	>50		0.056	positive
		multiple cropping index *X*_13_ (%)	≤180	(180, 200]	>200		0.043	positive
		percentage of agricultural GDP *X*_14_ (%)	≤20	(20, 30]	>30		0.046	positive
	drought resistance subsystem	GDP per capita *X*_15_ (Yuan per person)	≥15,000	[10,000, 15,000)	<10,000	0.238	0.030	negative
		percentage of reservoir regulation and storage *X*_16_ (%)	≥10	[5, 10)	<5		0.053	negative
		existing water supply capacity per unit area *X*_17_ (10^4^ m^3^ km^−2^)	≥100,000	[80,000, 100,000)	<80,000		0.038	negative
		percentage of effective irrigation area *X*_18_ (%)	≥0.85	[0.75, 0.85)	<0.75		0.045	negative
		emergency water supply capacity per unit area *X*_19_ (10^4^ m^3^ km^−2^)	≥4000	[3000, 4000)	<3000		0.028	negative
		monitoring and warning capacity *X*_20_ (station per 100 km^2^)	≥0.60	[0.40, 0.60)	<0.40		0.019	negative
		percentage of water-saving irrigation area *X*_21_ (%)	≥35	[25, 35)	<25		0.025	negative

* The larger the value of positive (negative) index, the higher (lower) the evaluation grade.

**Table 2:** The average connection number values and types of evaluation index for agricultural drought disaster risk in Suzhou from 2007 to 2017.

Evaluation System	Evaluation Subsystem	Evaluation Index	Connection Number Value		Semipartial Subtraction Set Pair Potential	
			Value	Type	Value	Status
agricultural drought disaster risk system	drought hazard subsystem	percentage of precipitation negative anomaly *X*_1_ (%)	0.416	weak promotion	0.388	partial identical potential
		annual precipitation *X*_2_ (mm)	−0.150	weak obstacle	−0.247	partial inverse potential
		relative humidity index *X*_3_ (%)	0.067	weak obstacle	0.013	symmetrical potential
		water resources amount per unit area *X*_4_ (10^4^ m^3^ km^−2^)	−0.441	middle obstacle	−0.485	partial inverse potential
		soil relative humidity *X*_5_ (%)	−0.105	weak obstacle	−0.205	partial inverse potential
		soil type *X*_6_	−0.170	weak obstacle	−0.207	partial inverse potential
	drought damage sensitivity subsystem	percentage of agricultural population *X*_7_ (%)	−0.627	strong obstacle	−0.712	inverse potential
		percentage of paddy field area *X*_8_ (%)	0.794	strong promotion	0.817	identical potential
		water consumption per RMB10^4^ GDP *X*_9_ (m^3^ per RMB10^4^)	0.057	weak obstacle	−0.021	symmetrical potential
		percentage of forest cover *X*_10_ (%)	0.395	weak promotion	0.400	partial identical potential
	exposure to drought subsystem	population density *X*_11_ (person km^−2^)	−0.632	strong obstacle	−0.691	inverse potential
		percentage of cultivated land *X*_12_ (%)	−0.597	middle obstacle	−0.608	inverse potential
		multiple cropping index *X*_13_ (%)	−0.231	middle obstacle	−0.321	partial inverse potential
		percentage of agricultural GDP *X*_14_ (%)	−0.054	weak obstacle	−0.132	symmetrical potential
	drought resistance subsystem	GDP per capita *X*_15_ (Yuan per person)	0.299	weak promotion	0.246	partial identical potential
		percentage of reservoir regulation and storage *X*_16_ (%)	−0.523	middle obstacle	−0.538	partial inverse potential
		existing water supply capacity per unit area *X*_17_ (10^4^ m^3^ km^−2^)	0.435	weak promotion	0.455	partial identical potential
		percentage of effective irrigation area *X*_18_ (%)	−0.642	strong obstacle	−0.691	inverse potential
		emergency water supply capacity per unit area *X*_19_ (10^4^ m^3^ km^−2^)	−0.172	weak obstacle	−0.194	symmetrical potential
		monitoring and warning capacity *X*_20_ (station per 100 km^2^)	−0.355	middle obstacle	−0.451	partial inverse potential
		percentage of water-saving irrigation area *X*_21_ (%)	−0.245	middle obstacle	−0.222	partial inverse potential

## Data Availability

The raw data supporting the conclusion of this article will be made available by the authors, without undue reservation.
